# Supplementary material for: Increasing population growth by asymmetric segregation of a limiting resource during cell division
Source: Mol Syst Biol. 2013 Apr 16;9:656. doi: 10.1038/msb.2013.13 (PMC3658268; doi:10.1038/msb.2013.13)
Supplement: Supplementary Information — Supplementary Figures S1–6 [file msb201313-s1.docx]

Increasing population growth by asymmetric segregation of a limiting resource

during cell division

Nurit Avraham, Ilya Soifer, Miri Carmi and Naama Barkai

*Department of Molecular Genetics, Weizmann Institute of Science, Rehovot 76100, Israel*

Correspondence: naama.barkai@weizmann.ac.il

Supplemental Inventory

# 1. Supplemental Figures

Supplementary Figure 1, related to Figure 1.

Supplementary Figure 2, related to Figures 1 and 2.

Supplementary Figure 3, related to Figure 3.

Supplementary Figure 4, related to Figure 5.

Supplementary Figure 5, related to Figure 5.

Supplementary Figure 6, related to the theoretical model.

# 2. Supplemental Movies

Supplementary Movie 1, related to Figure 1.

Supplementary Movie 2, related to Figure 5.

Supplementary Movie 3a, related to Figure 7.

Supplementary Movie 3b, related to Figure 7.

Supplementary Movie 3c, related to Figure 7.

# 3. Proposed models for asymmetric distribution of resources

**4. Supplemental Experimental Procedures**

**Strains**

**Time lapse microscopy**

**Flow cell**

**Growth conditions and cells preparation**

**5. Supplemental References**

1. Supplementary figures

**Supplementary figure 1**

1. ***The flow cell***. Flow cell setup used in the experiment. Cells are grown in a planar layer confined between a thin layer of PDMS, coated on a glass coverslip, and a permeable membrane. The medium is flowing above the membrane and is reaching the cell area by diffusion. The medium is switched automatically during the experiment through a computer-controlled device.
2. ***Linear growth curves***. Additional examples of linear growth curves in low zinc conditions measured in the flow cell. The two curves represent different experiments. For the blue curve, rich conditions were supplied at t=9h (red arrow). For the green curve the cells remained in low zinc till the end of the experiment. The slope depends on the number of mothers (dividing cells) in the population and therefore correlates with the initial number of cells that were shifted from rich to low zinc conditions at t=0h. Here the mother cells continued to divide for about 8 more generations with an averaged doubling time of 120±15 min.
3. ***Linear growth curves in batch cultures***. Linear growth curves measured in batch cultures in two different experiments. Wild type cells where grown over night to OD_600_ = 0.5. The cells then washed twice in low zinc media, diluted to OD_600_ = 0.1, and their OD was measured as a function of time.
4. ***Equivalent protein expression in mothers and daughters***. Cells were transferred to low zinc, as described above, and the expression of the high affinity zinc transporter Zrt1 was followed as a function of time, using a fluorescent reporter (the fusion protein, Zrt1-mChery). Each curve represents a single cell that is either a mother (blue) or a daughter (red). The inset shows the distribution of fluorescence in the two subpopulations.

**Supplementary figure 2**: **Mother only proliferation in low metal conditions**

To verify that the transition into linear growth with mother only proliferation results from the change in metal status and not from other factors, we performed the same experiment as in Figures 1 and 2 where we shift the cells from rich to poor medium but this time we used for the rich medium the low metal media with supplement of the appropriate metal:

1. (b) Linear growth (a) and splitting into mothers and daughters (b) for cells transferred from LZM + 300 µM Zn^2+^ to LZM +10 µM Zn^2+^ (low zinc medium).
2. (d) Linear growth (c) and splitting into mothers and daughters (d) for cells transferred from low iron medium + 10 µM FeCl_3_ to a low iron medium.
3. (f) Linear growth (e) and splitting into mothers and daughters (f) for cells transferred from low copper medium + 10 µM Cu to low copper medium.

**Supplementary figure 3**. ***Growth curves of whi5 in low zinc***. Growth curves of *whi5* cells measured in the flow cell in low zinc conditions. Note the saturation in growth after ~ 18 hours.

**Supplementary Figure 4**

1. When growing wild type cells in semi-low conditions the population exhibits unimodal size distribution, indicating uniform behavior of the population, with no splitting into subpopulation
2. When growing *vac17* cells in semi-low conditions, the population exhibits bimodal size distribution, manifesting the observed splitting into mothers and daughters.
3. Shown is a sequence of snapshots of Supplementary Movie 2, presenting *vac17* cell, grown in semi-low zinc conditions, giving rise to four daughter cells that do not divide.


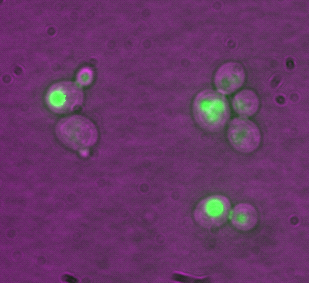


b

Vacuolar marker

Zinc marker


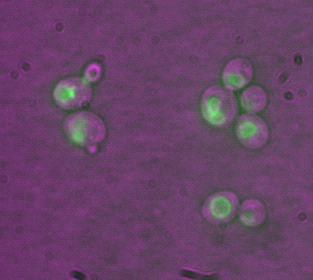


a

**Vacuolar marker (Vph1-GFP) Zinc marker (Fluozin-1)**

**Supplementary Figure 5**. ***Vacular zinc accumulation.***

Wild type cells containing vacuolar marker (fusion protein *vph1*-gfp) were grown in low zinc for 6 hours, and then were treated by FuraZin-1 as described in (MacDiarmid *et al*, 2003), in order to mark the zinc inside the cell and visualize zinc distribution inside the cells. Shown are fluorescent images of the cells vacuoles (a) and the distribution of zinc inside the cell (b). Note that most liable zinc is located inside the vacuoles.

1. Supplementary Movies

# Supplementary Movie 1 -

Splitting into dividing mothers (blue) and non-dividing daughters (red).

**Supplementary Movie** **2**

A *VAC17* deleted cell, defective in vacuole inheritance, grown under semi-limited conditions gives rise to four non dividing daughter cells.

# Supplementary Movie 3a -

Cells (mostly daughters) imploded during osmotic stress.

# Supplementary Movie 3b -

An example of a mother cell (blue) giving birth to daughter cells (red) that die immediately, while the mother continues to divide.

# Supplementary Movie 3c -

Zoom in on a region of a colony during its recovery from rapamycin. Mothers are exploding and dying (marked by disappearance of their blue numbers) while daughters start to divide (the newborn cells are marked in green).

1. Proposed models for asymmetric distribution of resources.

In this section we formulate and analyze a quantitative model explaining the potential benefit of asymmetric distribution of resources for increasing overall population growth under limiting conditions. We first provide intuition by considering a simple model, as discussed in main text. The idea is simply that if some level of a limiting resource is required for division, preventing its dilution within one progenitor may allow the division of this progenitor for a very long time, while division will halt quickly if the resource will be split at each division. Second, we extend the model to a more realistic setting and solve this extended model using numerical simulations to show that it can explain our experimental observations.

**Core Model:**

We consider cells that are transferred to a condition where their ability to produce/accumulate a limiting resource Z is limited. Our core model is based on the following assumptions:

1. Prior to the shift, cells possess a quantity Z_0_ of the limiting resource.

2. In the limiting conditions, cells utilize a$\Delta Z>0$amount of resource during each cell cycle.

3. A cell requires a minimal amount of the limiting resource, Z_c_ in order to divide.

Our goal is to compare the overall population growth under symmetric vs. asymmetric division. To this end, we first consider two limiting cases. First, if the available Z is split equally between the two daughter cells, its level in each cell division is reduced as:

$Z^{\left( n+1 \right)}=\frac{1}{2}\times\left( Z^{\left( n \right)}-\Delta Z \right)$, Z ^(0)^ = Z_0_

where $Z^{\left( n \right)}$ denotes the level of limiting resource in the cell at the n^th^ generation. Division will halt after *n_s_* generations, for which $Z^{{(n}_{s})}\leq Z_{c}$. The total number of cells when the division halts is then: $N_{s}=2^{n_{s}}\leq\frac{Z_{0}+\Delta Z}{Z_{c}+\Delta Z}$

Second, if the limiting resource is maintained only in one of the progenitors, the decay in its level will be given by:

$Z^{\left( n+1 \right)}=1\times\left( Z^{\left( n \right)}-\Delta Z \right),$ Z^(0)^ = Z_0_

In this case, division will halt after *n_as_* number of generations, again defined by $Z^{(n_{as})}\leq Z_{c}$, giving rise to a total of progenies:

$$N_{as}=n_{as}\leq\frac{{Z_{0}-Z}_{c}}{\Delta Z},$$

Clearly, under a broad set of parameters Z_0_, Z_c_, ΔZ the asymmetrically-dividing population will reach a higher final size compared to the symmetrically dividing population *N_as_* > *N_s_*. This is most easily understood by considering the cases where the inequalities are saturated.

Extending the model:

To better examine the ability of the model to qualitatively explain our data, we extended it to include three additional features that were not considered in the core model:

1. We allow accumulation of the limiting factor during the cell cycle. That is, we assume that *Z* is slowly increasing, $\dot{Z}>0$.

2. We allow for different levels of asymmetric segregation. To this end, we introduce an additional parameter *α* which defines the fraction of limiting resource maintained in the mother cell (*α* = 0.5 corresponds to the fully symmetric case, while α=1 is the fully asymmetric case).

3. We allow stochasticity in the cell-division threshold. Thus, rather than assuming that division is prohibited for cells with *Z* < *Z*_C_, we assume that division can occur with some probability that depends on the level of cellular *Z*. As a concrete form of this probability we consider:

$$p\left( Z,\Delta t \right)=\left\{ \begin{aligned} 0 \Delta t<T_{ab} \\ c\times(1+{tanh((Z-Z_{c})}/{\delta Z})) \Delta t>T_{ab} \end{aligned} \right.$$

where Δt is the cell ‘age’, the time that had elapsed since the last division. In this function, the constants δZ and *c* determine the width of the doubling time distribution. If δZ is much smaller than all other constants, then the division probability has a sharp threshold at Z_c_.

The dynamics is therefore simulated as follows: We begin with a progenitor cell that has some initial level of resources Z_0_*_._* At any time point, the cell can divide with a probability given by *p*(*Z*, ∆t), depending on its internal resource level and the age. Once a cell divides, the resource is distributed between mothers and daughters, where the mother receives a fraction α of the available resource and the daughter receives a fraction (1- α). In addition, the level of resource in each cell is continuously increasing at a fixed rate, $\dot{Z}=\beta$. Note that this parameter defines the difference between rich and limiting conditions: rich conditions correspond to a high *β*, while poor conditions correspond to low *β*. The number of cells in the system is recorded throughout the simulation.

Supplementary Figure 6 shows simulated growth curves, comparing the symmetric and the asymmetric cases. In poor conditions, asymmetric distribution of resources results in a faster growth compared to symmetric case (Supplementary Figure 6d and 6f), while in rich conditions, symmetric resource distribution is more beneficial (Supplementary Figure 6c and 6e). This conclusion holds for different forms of the division probability *P* approximating either the deterministic (δZ << Z_C_ and c = 0.5, corresponding to a sharp threshold for initiating division, Figure 6e and 6f) or the stochastic division (Figure 6c and 6d).

**Supplementary Figure 6:** **Stochastic model for growth with asymmetric distribution of resources.**

1. *Symmetric distribution of resources*. A mother cell (blue) with internal amount $Z$ divides into mother and daughter with $\frac{1}{2}Z$. The time until the cell is ready to divide again ($Z>Z_{c})$is determined by $Z$.
2. *Asymmetric distribution of resources.* Most of the resource is retained by the mother ($\frac{9}{10}Z$), while the daughter (red) gets only small amount $\frac{1}{10}Z$ or no resource at all. The mother is rapidly ready to divide again while the daughter remains undivided.
3. (d) *Growth dynamics in rich and poor conditions.*
   C=0.035, $\delta Z=1.5$, *T_ab_*=90, Z_c_ = 45 (c) Growth curves for the symmetric and asymmetric cases in rich conditions ($\beta=0.3)$. As expected, the population grows faster for symmetric distribution of resources. (c) Same for poor conditions ($\beta=0)$. In the symmetric case the growth decays at some point, while in the asymmetric case (α=0), the population grows indefinitely.
4. (f) Growth *dynamics in rich and poor conditions – deterministic case.*c = 0.5, $\delta Z=0.005$, *T_ab_*=90 minutes, Z_c_ = 45, Z_0_ = 200. (e) Growth curves in rich conditions ($\beta=0.3)$ for the symmetric and asymmetric cases (α=0), starting from a single cell. As expected, the population grows faster for the symmetric case. (f) Same for poor conditions ($\beta=0)$. Here, in the symmetric case, the population stops dividing after 8 generations, while in the asymmetric case (α=0), the population grows indefinitely.
5. Supplemental experimental procedure

**Strains**

The wild type strain is based on Y8205(Tong and Boone, 2006), and contains fusions of eGFP and mCherry to CDC10 and ACS2 respectively. Deletion strains with fluorescent markers were made by transformation or by mating the wild type strain with the corresponding a type strain from the yeast deletion collection (EUROSCARF) (Giaever *et al*, 2002) and subsequent sporulation and selection for the alpha type haploids using the SGA method.

| Zrc1-mCherry | BY4741 *CDC10-GFP::HIS3, ZRC1-mCherry::hph* | Fusion of mCherry::hph downstream of *ZRC1* ORF in strain CDC10-GFP::HIS3 from GFP library (Huh *et al*, 2003) |
| --- | --- | --- |
| Zrt1-mCherry | BY4741 *CDC10-GFP::HIS3, ZRT1-mCherry::hph* | Fusion of mCherry::hph downstream of ZRT1 ORF in strain CDC10-GFP::HIS3 from GFP library (Huh *et al*, 2003) |
| Vph1-mCherry | BY4741 *VPH1-mCherry::hph* | CDC10-GFP::HIS3 from GFP library(Huh *et al*, 2003) |
| *whi5*-*vph1* | *BY4741 whi5*Δ*::kanMX VPH1-mCherry::hph* | Fusion of mCherry::hph downstream of VPH1 ORF into  *whi5* from deletion library (Giaever *et al*, 2002) |
| *vma16* | *vma16*Δ:*:kanMX* | From deletion library (Giaever *et al*, 2002) |
| *zrc1* | *zrc1*Δ:*:kanMX* | From deletion library (Giaever *et al*, 2002) |
| *zrc1-cot1* | *BY4741 hoΔ::natMX-TEF2pr-GFP zrc1Δ::his3 cot1Δ::kanMX* | Transformation of BY4741 |

**Time lapse microscopy**

Growth of microcolonies was observed with ﬂuorescence time-lapse microscopy at 30 C° using an Olympus IX-81-ZDC inverted microscope with a motorized stage (Prior). Image sets were acquired with a Hamamatsu ORCA-II-BT camera. Fluorescent proteins were detected using EXFO X-Cite light source and Chroma 49002 ET-GFP and 49008 ET-mCherry filter sets. Cells were observed in a microfluidic flow cell (Supplementary Figure 1a) connected to two micro-perfusion pumps, that enabled to grow the cells in a planar layer while simultaneously controlling their extracellular environment. ImagePro 6.3.1 (Media Cybernetics) was used to automate image acquisition, microscope control and pumps control.

**Flow cell**

In order to grow the cells in a planar layer while simultaneously control their extra cellular environment, we used a standard FCS2 Flow cell (Bioptechs) and improved it in the following way (Charvin *et al*, 2008): the 40 mm round coverslips were coated with a thin layer of PDMS (Sylgrad 184,GE). This was done in a clean room, using a suitable spinning procedure to achieve layers of ~ 30 µm thick. To confine the cells and prevent their movement, while medium is flowing through the chamber, we used a diffusive cellulose membrane, which was cut from dialysis tubes (Sigma Aldrich, D9527). The membrane was cleaned and prepared as described in (Charvin *et al*, 2008). As shown in Supplementary Figure 1, the cells are confined between the PDMS layer and the membrane, while the medium is flowing above the membrane. In this way the medium reaches the cells area without exerting too much force on the cells. The flow cell is then connected to two micro-perfusion pumps (Instech), which are controlled automatically by the computer via a D/A convener (measurement computing usb-3110), enabling to change the medium during the experiment without perturbing the cells.

**Growth conditions and cells preparation**

Low zinc media (LZM) was prepared as described in (Zhao and Eide, 1996), and had the following composition: 0.17% yeast nitrogen base without amino acids, (NH_4_)_2_SO_4_, or zinc (BIO101); 0.5% (NH_4_)_2_SO_4_; 10 mM trisodium citrate, pH 4.2; 2% glucose; 1 mM Na_2_ EDTA; and 1% adenine, histidine, leucine, and tryptophan. The MnCl_2_ and FeCl_3_ concentrations in LZM were adjusted to final concentrations of 25 and 10 µM.

In a typical experiment the cells were pre-grown for around 24 hours in SC medium to OD_600_ of about 0.5. A droplet of log-phase cells (5.3 µl at OD_600_=0.2) was then deposited onto a coated coverslip and was covered by a wet membrane. Before shifting to LZM, the cells were allowed to adjust to the environment of the flow cell for an hour, while non limiting medium is flowing through the flow cell. The medium was then switched to a low zinc medium (LZM) and a set of bright field, red-fluorescence and-green fluorescence images was acquired every 6 minutes, for 6 different fields of view. In some experiments the cells were pre-grown in a semi-limited environment (50% SC, 50% LZM) for 4 hours before loaded into the flow cells. This treatment was found to accelerate the splitting, to decrease the number of generations before halting and to increase the doubling time of the dividing mothers; when transformed from SC to LZM the splitting occurred after 3 hours, the mothers continued to divide for 10 more generations on average, and the average doubling time was 120 min. when the cells were grown in semi-limited conditions prior to the shift, the splitting occurred after 2 hours, the mothers divided for 5 more generations on average, and the average doubling time was 150 min.

**Measurement of Vacuole Zinc Content with FuraZin-1**

Yeast cultures (250 ml) were grown to log phase in LZM + 2 µM zinc. Cells were

harvested, washed twice with phosphate-buffered saline and resuspended in phosphate-buffered saline at a final density of 3X10^8^ cells/ml. 50 µg of FuraZin-1 acetoxymethyl (AM) ester was dissolved in16.6 µl of a 20% Pluronic solution (both obtained from Molecular Probes) and the solution diluted 4-fold with Me_2_SO to give a stock solution of 1.25 mM fluorophore and 5% Pluronic. Fluorophore was added to a 1-ml aliquot of the cell suspensions to give a final concentration of 25 µM. Another 1-ml aliquot of each strain was treated in parallel but without exposure to fluorophore. The cell suspensions were incubated at 30 °C in the dark for 1 h with agitation. The cells were recovered by centrifugation, washed three times with 5 ml of chilled

zinc uptake buffer (10 mM MES-Tris, pH 6.5, 4 mM MgCl2, 2% glucose) and 1 mM EDTA and then incubated at 30 °C for another 30 min. This step allowed the cells to redistribute cytoplasmic fluorophore to the vacuole and complete its hydrolysis. The cells were then chilled and washed twice with MES-Tris uptake buffer (-EDTA). The cell were then measured under the microscope.

.

1. Supplemental references

Charvin G, Cross FR, Siggia ED (2008) A microfluidic device for temporally controlled gene expression and long-term fluorescent imaging in unperturbed dividing yeast cells. *PLoS One* **3:** e1468.

Giaever G, Chu AM, Ni L, Connelly C, Riles L, Veronneau S, Dow S, Lucau-Danila A, Anderson K, Andre B, Arkin AP, Astromoff A, El-Bakkoury M, Bangham R, Benito R, Brachat S, Campanaro S, Curtiss M, Davis K, Deutschbauer A, Entian KD, Flaherty P, Foury F, Garfinkel DJ, Gerstein M, Gotte D, Guldener U, Hegemann JH, Hempel S, Herman Z, Jaramillo DF, Kelly DE, Kelly SL, Kotter P, LaBonte D, Lamb DC, Lan N, Liang H, Liao H, Liu L, Luo C, Lussier M, Mao R, Menard P, Ooi SL, Revuelta JL, Roberts CJ, Rose M, Ross-Macdonald P, Scherens B, Schimmack G, Shafer B, Shoemaker DD, Sookhai-Mahadeo S, Storms RK, Strathern JN, Valle G, Voet M, Volckaert G, Wang CY, Ward TR, Wilhelmy J, Winzeler EA, Yang Y, Yen G, Youngman E, Yu K, Bussey H, Boeke JD, Snyder M, Philippsen P, Davis RW, Johnston M (2002) Functional profiling of the Saccharomyces cerevisiae genome. *Nature* **418:** 387-391.

Huh WK, Falvo JV, Gerke LC, Carroll AS, Howson RW, Weissman JS, O'Shea EK (2003) Global analysis of protein localization in budding yeast. *Nature* **425:** 686-691.

MacDiarmid CW, Milanick MA, Eide DJ (2003) Induction of the ZRC1 metal tolerance gene in zinc-limited yeast confers resistance to zinc shock. *J Biol Chem* **278:** 15065-15072.

Tong AH, Boone C (2006) Synthetic genetic array analysis in Saccharomyces cerevisiae. *Methods Mol Biol* **313:** 171-192.

Zhao H, Eide D (1996) The ZRT2 gene encodes the low affinity zinc transporter in Saccharomyces cerevisiae. *J Biol Chem* **271:** 23203-23210.
